# Effects of Blast Overpressure on Neurons and Glial Cells in Rat Organotypic Hippocampal Slice Cultures

**DOI:** 10.3389/fneur.2015.00020

**Published:** 2015-02-12

**Authors:** Anna P. Miller, Alok S. Shah, Brandy V. Aperi, Matthew D. Budde, Frank A. Pintar, Sergey Tarima, Shekar N. Kurpad, Brian D. Stemper, Aleksandra Glavaski-Joksimovic

**Affiliations:** ^1^Department of Neurosurgery, Medical College of Wisconsin, Milwaukee, WI, USA; ^2^Department of Cell Biology, Neurobiology and Anatomy, Medical College of Wisconsin, Milwaukee, WI, USA; ^3^Clement J. Zablocki Veterans Affairs Medical Center, Milwaukee, WI, USA; ^4^Division of Biostatistics, Institute for Health and Society, Medical College of Wisconsin, Milwaukee, WI, USA

**Keywords:** blast injury, traumatic brain injury, *in vitro* model, organotypic slice culture, hippocampus, cell death

## Abstract

Due to recent involvement in military conflicts, and an increase in the use of explosives, there has been an escalation in the incidence of blast-induced traumatic brain injury (bTBI) among US military personnel. Having a better understanding of the cellular and molecular cascade of events in bTBI is prerequisite for the development of an effective therapy that currently is unavailable. The present study utilized organotypic hippocampal slice cultures (OHCs) exposed to blast overpressures of 150 kPa (low) and 280 kPa (high) as an *in vitro* bTBI model. Using this model, we further characterized the cellular effects of the blast injury. Blast-evoked cell death was visualized by a propidium iodide (PI) uptake assay as early as 2 h post-injury. Quantification of PI staining in the cornu Ammonis 1 and 3 (CA1 and CA3) and the dentate gyrus regions of the hippocampus at 2, 24, 48, and 72 h following blast exposure revealed significant time dependent effects. OHCs exposed to 150 kPa demonstrated a slow increase in cell death plateauing between 24 and 48 h, while OHCs from the high-blast group exhibited a rapid increase in cell death already at 2 h, peaking at ~24 h post-injury. Measurements of lactate dehydrogenase release into the culture medium also revealed a significant increase in cell lysis in both low- and high-blast groups compared to sham controls. OHCs were fixed at 72 h post-injury and immunostained for markers against neurons, astrocytes, and microglia. Labeling OHCs with PI, neuronal, and glial markers revealed that the blast-evoked extensive neuronal death and to a lesser extent loss of glial cells. Furthermore, our data demonstrated activation of astrocytes and microglial cells in low- and high-blasted OHCs, which reached a statistically significant difference in the high-blast group. These data confirmed that our *in vitro* bTBI model is a useful tool for studying cellular and molecular changes after blast exposure.

## Introduction

The incidence of blast-induced traumatic brain injury (bTBI) has escalated dramatically as the use of improvised explosive devices (IEDs) and improvised rocket assisted mortars (IRAMS) has risen during current military conflicts (1–4). Severity of bTBI can range from mild to severe, with a wide variety of symptoms producing physical, cognitive, and emotional consequences (5–10). The combination and severity of symptoms is dependent on patient and exposure characteristics, which tend to vary significantly in the field. Therefore, characterizing the human time course of post-injury symptomatology and healing is complicated by the variability in patient outcomes. Confounding the issue is that bTBI patients may often be incorrectly diagnosed with post-traumatic stress disorder (PTSD) (4, 5, 11). Accordingly, the quality of life for victims of bTBI is substantially decreased and the effectiveness of currently available treatment protocols is limited.

While penetrating and blunt injury mechanisms from secondary (objects propelled by the blast) and tertiary (individuals being thrown by the blast wind) blast effects are well understood (12, 13), mechanisms of cellular brain damage following primary blast exposure remain unclear. Various theories for the cause of neuronal damage during bTBI have included blast wave propagation via thoracic mechanisms, ischemic brain damage, head acceleration, and direct skull deformation (14–20). However, studies from our group together with studies from other laboratories suggest that the blast shockwave directly penetrates the cranium and transverses brain tissues, resulting in mechanical strain-induced damage (21–25). Recent studies in *in vitro* bTBI models, without confounding *in vivo* factors, also demonstrated a direct effect of blast overpressure on SH-SY5Y human neuroblastoma cells (26, 27) and organotypic hippocampal slice cultures (OHCs) (28, 29).

The precise mechanisms of neuronal damage during blast exposure remain elusive, and clinical evidence suggests that they are distinct from mechanisms of closed head (blunt) and penetrating TBI (5, 30). Immediately following the initial brain tissue insult, the damaged area can undergo ischemia, edema, vasoconstriction, inflammation, and accumulation of free radical oxygen species, excitatory amino acids (EAA), or certain ions (5, 8, 21, 22, 31). Similar to non-blast TBI, this cascade of secondary events results in further neuronal degeneration and death that is characterized at the ultrastructural level by swollen neurons with pyknotic nuclei, darkened atrophic dendrites, and axonal injury characterized by axonal varicosities and disruption of axonal transport (15, 20, 32–36). Besides those neuronal effects, blast waves also caused astrocyte and microglial activation consistent with the activation of inflammatory processes and oxidative stress (20, 32, 36–40). Although the above effects on neurons and glial cells were observed following blast exposure, it is still unknown whether they are achieved directly by the blast overpressure or through indirect mechanisms.

Understanding the cellular and molecular cascade of events involved in neurodegeneration following bTBI is essential for the development of effective treatments. Due to complex neuropathology, assessments of potential novel treatments require an experimental model that is easily manipulated, but sufficiently complex to resemble the *in vivo* situation. To meet this challenge and to investigate direct effects of blast exposure on neuronal and glial cells without confounding *in vivo* factors, we have generated an *in vitro* bTBI model utilizing OHCs. OHCs provide remarkable advantages over research conducted in monolayer culture models since they retain three-dimensional tissue-specific cytoarchitecture with appropriate neuronal–glial interaction and neuronal circuits (41–44). Though monolayer neuroblastoma and glial cell cultures have provided insight into the cellular attributes of blast damage (26, 27, 45), they fail to model the complex heterogeneous organization found in living tissue that is essential for understanding the neurodegenerative consequences of blast injury. Importantly, OHCs have been successfully used to model different diseases and as screening platforms for novel therapeutic approaches (46–50). For our study, we used hippocampal tissue since it has been shown that hippocampal neurons are highly susceptible to blast injury (15, 20, 25, 28, 39, 40, 51) as well as to non-blast TBI (52–57), and hypoxic and ischemic conditions (58, 59). Given the importance of the hippocampus to learning and memory (60, 61), understanding the cellular and molecular events associated with the hippocampal blast injury are essential in understanding trauma pathology and development of novel treatment strategies.

In this report, an open-ended, helium-driven shock tube was used to expose OHCs to blast overpressures of 150 and 280 kPa, simulating a blast injury. Blast-evoked cell death was assessed by propidium iodide (PI) uptake and lactate dehydrogenase (LDH) release assays. To further characterize the effects of blast exposure on neurons and glial cells, immunohistochemical staining was performed.

## Materials and Methods

### Animals

Pregnant Sprague-Dawley (SD) rats were obtained from Charles River Laboratories (Wilmington, MA, USA) and were housed through parturition in individual cages under standard colony conditions with food and water available *ad libitum*. Brain tissue for hippocampal slice culture preparation was harvested from postnatal pups (P7–P10; *n* = 51). All animal-related procedures were conducted in accordance with NIH Guide for the Care and Use of Laboratory Animals and approved by the Zablocki Veterans Affairs Subcommittee for Animal Studies.

### Preparation of OHCs

Organotypic hippocampal slice cultures were prepared under sterile conditions using a slightly modified method from Stoppini et al. (41). The postnatal SD rats were sacrificed by decapitation and the skulls opened longitudinally along the midline. Brains were aseptically removed and placed in cold dissecting medium (pH 7.2) containing 50% minimum essential medium (MEM), 50% calcium and magnesium free Hank’s Balanced Salt Solution (HBSS), 20 mM HEPES (*N*-2-hydroxyethylpiperazine-*N*′-2-ethanesulfonic acid), 7.5 g/l d-glucose, and 1% penicillin/streptomycin (all obtained from GIBCO Life Technologies, Grand Island, NY, USA) (62). The hippocampi were dissected and transversely cut into 400 μm sections using a McIlwain tissue chopper (Ted Pella, Inc., Redding, CA, USA). Hippocampal sections were transferred to a dish containing dissecting medium and were carefully separated under a dissecting microscope using a pair of sterile spatulas. Only sections with the intact morphology were transferred to 0.4 μm MilliCell cell culture inserts (Millipore, Billerica, MA, USA) and deposited into 6-well plates. Four to six slices were placed on each insert and maintained in 1 ml of serum-based media consisting of 50% MEM-Hank’s medium, 25% HBSS, 25% horse serum, 50 mM HEPES, 2 mM l-glutamine, 5 mg/ml d-glucose, and 1% antibiotic/antimycotic (all obtained from GIBCO Life Technologies) (63–65).

Throughout the duration of the experiment, OHCs were maintained at 37°C in 5% CO_2_. The culture medium was changed the day after preparation of slices. From 4 to 7 days *in vitro* (DIV), serum-based culture medium was gradually changed to a serum-free medium consisting of 50% MEM-Hank’s, 25% HBSS, 25% Neurobasal-A medium, 17 mM HEPES, 2 mM l-Glutamine, 2% B-27, 5 mg/ml d-glucose, and 1% antibiotic/antimycotic (all obtained from GIBCO Life Technologies). From 7 DIV until the end of the experiment, OHCs were grown in a serum-free medium (64, 66, 67) to decrease astrocyte proliferation and microglial activation (68, 69). All PI uptake and LDH release measurements, described below in more detail, were also performed in the serum-free medium due to the interference of serum with these cell viability assays (70, 71).

### Shock tube

**Figure 1:**
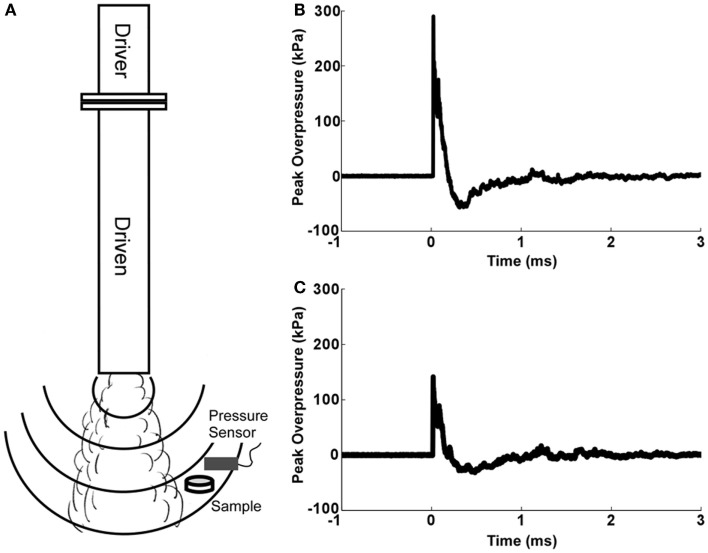
**Open-ended helium-driven shock tube**. **(A)** The shock tube consists of driver and driven sections separated by a Mylar membrane that bursts at a specific pressure to create a blast overpressure of a predetermined magnitude. For blast injury, culture dishes containing serum-free medium and Millicell inserts with OHCs were sealed inside sterile plastic pouches and placed below the tube and out of the blast wind. Peak overpressures were recorded by pressure sensor placed above the culture dish with OHCs. **(B)** Representative pressure profile from OHCs exposed to high-blast overpressure (~280 kPa). **(C)** Representative pressure profile from OHCs exposed to low blast overpressure (~150 kPa).

Our group has designed a compressed gas-driven, open-ended shock tube (Figure 1), which was used to expose OHCs to shockwave overpressure. The 7.5 cm-diameter, vertically oriented shock tube consists of a 17-cm driver section and a 152-cm driven section separated by a Mylar diaphragm. The driver section was pressurized with helium gas until exceeding the bursting pressure, at which time a shockwave was formed and traveled down the length of the driven section. Thickness of the Mylar membrane controlled bursting pressure and shockwave overpressure magnitude.

Organotypic hippocampal slice culture specimens were rigidly positioned off axis to ensure shockwave exposure without the disruptive mechanical effects of the blast wind. Exposures demonstrated very high repeatability across all samples (coefficient of variation <10%).

### Blast injury

**Figure 2:**
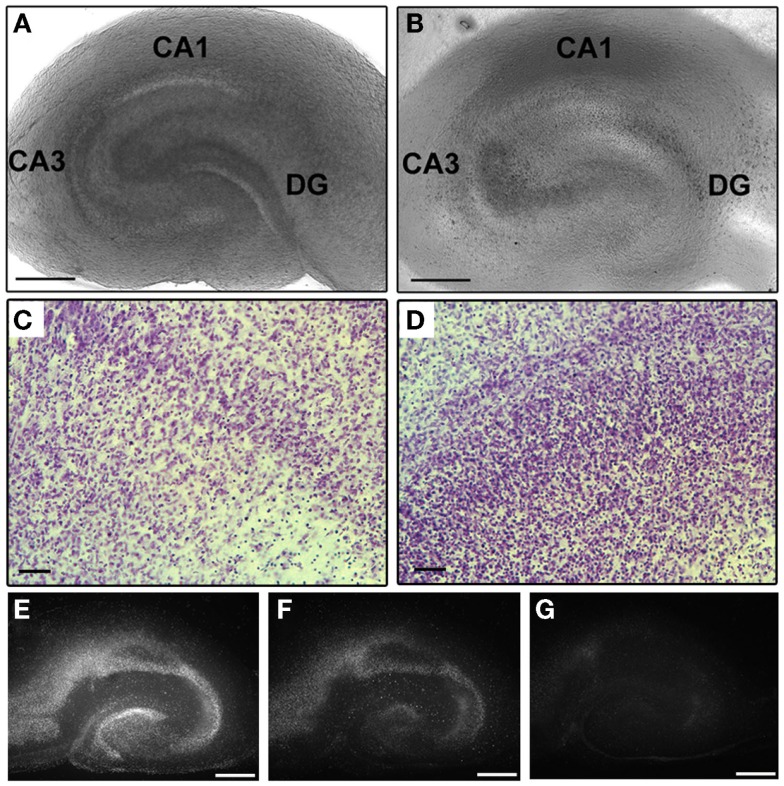
**Preservation of OHCs’ structural organization during culturing period**. **(A)** Light micrograph of an acutely dissected OHC. **(B)** Same OHC as in **(A)** demonstrates well preserved CA1, CA3, and DG hippocampal regions at 8 DIV. Higher magnification of CA1 **(C)** and DG **(D)** regions of cresyl violet-stained OHC at 8 DIV also illustrate intact hippocampal cytoarchitecture. Serial imaging of PI-stained OHC at 1 **(E)**, 5 **(F)**, and 8 **(G)** DIV demonstrates recovery of slice from procedure-related cellular degeneration. Scale bars **(A,B)** 500 μm; **(C,D)** 50 μm; **(E–G)** 500 μm.

Organotypic hippocampal slice cultures were grown for 8 days prior to blast exposure. Our data (Figure 2) together with the data from other groups (72, 73) demonstrated that this period is sufficient to allow slice procedure-related cellular degeneration to end. In addition, it has been demonstrated that by 7 DIV the majority of microglial cells return to the resting, ramified phenotype (74–76). At 8 DIV, OHCs were exposed to blast injury. Individual inserts with 4–6 OHCs were placed in 40-mm culture dishes containing 800 μl of serum-free medium, covered with Parafilm and sealed inside sterile plastic pouches (5 cm × 6.5 cm). Samples were placed on a rigid holder below the shock tube at 55° off axis. The distance from the end of the shock tube to the cultures was 22 cm. Samples were exposed to a single blast overpressure of 147 ± 18 kPa (low) or 278 ± 22 kPa (high). Side-on pressure was recorded using PCB113B28 (PCB Piezotronics, Depew, NY, USA) sensors located directly above the test sample at 10 MHz (National Instruments, Austin, TX, USA). The average duration and impulse for the low blast overpressure were 160.3 ± 15.7 μs and 10.39 ± 1.23 kPa × ms. The corresponding values for the high-blast group were 157.1 ± 8.2 μs and 18.10 ± 1.88 kPa × ms. Following blast exposure, under sterile conditions, inserts with OHCs were removed from the pouches and placed back in the incubator in fresh serum-free medium. Four different control groups were included in the studies. Incubator controls remained in the incubator throughout experimentation. Sham-exposed OHCs were prepared using an identical protocol, placed under the shock tube, but not exposed to the shockwave overpressure. In addition, low- and high-vibration control groups were used to determine the effect of mechanical vibration due to the firing of the shock tube. Vibration control OHCs were prepared using an identical protocol and placed on a separate rigid holder below the tube. This second holder was attached to the shock tube system, but located away from the shockwave. These OHCs were exposed to the system vibration, but not to the blast overpressure. Cell death attributable to mechanical vibration was quantified by comparing the vibration control group to the sham control group.

### Assessment of cell death with PI uptake

The red fluorescent dye, PI (GIBCO Life Technologies), was used as a marker for cell death, as it only enters into cells with damaged cell membranes (77). Two hours (h) before imaging, PI was added to the OHC culture medium at a final concentration of 2 μM (78, 79). PI fluorescence emission was assessed prior to (0 h) and at 2, 24, 48, and 72 h following blast exposure with a Nikon Eclipse TE2000-U upright fluorescent microscope (Nikon Instrument Inc., Melville, NY, USA) at the 4× objective. Damaged OHCs with a high number of PI-stained cells at 0 h were excluded from subsequent studies. A digital SPOT camera and software (Spot Imaging Solutions, Sterling Heights, MI, USA) were used to capture all images under identical conditions. Digitized 12-bit images were used to quantify PI staining in the outlined cornu Ammonis 1 and 3 (CA1, CA3), and dentate gyrus (DG) subfields of each section using custom MATLAB software (Mathworks, Inc., Natick, MA, USA). Images were corrected for non-specific background staining by applying a top-hat filter followed by thresholding (80, 81). The threshold was established based on MATLAB generated histograms of PI pixel intensity values for all OHCs at the 0 h and the same threshold was applied to all images (29, 82). The cell death was quantified as the percent area of staining above the threshold within the region of interest (ROI) (29, 80, 81, 83).

### Assessment of cell death with LDH assay

At 2, 24, 48, and 72 h post-injury, the total volume of culture medium from wells containing 5 OHCs was collected and stored at −80°C until analysis. Quantification of LDH release into the culture medium, which is directly proportional to the cell death (44, 78), was performed using a Cytotoxicity LDH Detection kit (Clontech Laboratories Inc., Mountain View, CA, USA), according to the manufacturer’s protocol. Culture medium from each well was centrifuged at 1200 rpm for 10 min and 100 μl of supernatant was combined with 100 μl of reaction mixture. Following incubation at room temperature for 30 min, the absorbance was read at 490 nm with a reference wavelength of 600 nm using a microplate reader (PowerWave XS, BioTek Instruments Inc., Winooski, VT, USA) (44, 84). Analysis was run in triplicate for each well. LDH activity was quantified using a standardized curve of known concentrations (78). Culture medium collected from 5 to 8 wells per group was analyzed at each time point.

### Cresyl violet staining

At 8 DIV, cresyl violet staining was performed using a slightly modified protocol published by Cho et al. (85). OHCs were fixed at room temperature with 4% paraformaldehyde (PFA) in 0.1 M phosphate-buffer (PB; pH 7.4) for 30 min and processed for staining on the insert membrane. After fixation, OHCs were washed three times for 5 min in phosphate buffered saline (PBS, pH 7.4). OHCs were then immersed in 0.75% cresyl violet solution (Sigma Aldrich, St. Louis, MO, USA) for 7 min. After rinsing in distilled water for 30 s, OHCs were dehydrated in a graded series of ethanol (70, 90, 95, and 100%) for 1 min at each concentration, and cleared in xylene (two times for 10 min). Stained OHCs were mounted between microscope slides and coverslips using DPX mounting medium (Sigma Aldrich). Light microscope images were obtained using a Nikon E600 epifluorescent microscope equipped with the Sight DS-Fi1 digital color camera (Nikon Instrument Inc.).

### Immunohistochemistry

At 72 h post-injury, OHCs were fixed with 4% PFA in 0.1 M PB for 30 min at room temperature. Immunostaining was preformed directly on the insert membranes that were cut out around the OHCs (85–87), except immunostaining against neuronal class III β-tubulin (Tuj-1), which was done on free floating sections (88). Following fixation, OHCs were washed 3 × 5 min in PBS, and placed for 1 h in blocking solution containing PBS, 1% Triton-X 100 (Sigma Aldrich), 5% normal goat serum (NGS; GIBCO Life Technologies), and 5% bovine serum albumin (BSA; Sigma Aldrich). Primary antibodies were diluted in a blocking solution as follows: polyclonal rabbit anti-glial fibrillary acidic protein (GFAP; Dako, Carpinteria, CA, USA) 1:500, polyclonal rabbit anti-ionized calcium-binding adapter molecule 1 (Iba1; Abcam, Cambridge, MA, USA) 1:200, and polyclonal rabbit anti-Tuj-1 (Covance, Princeton, NJ, USA) 1:100. Incubation with primary antibodies was performed in a humid atmosphere at 4°C for 48 h. After washing, primary antibody–antigen complexes were visualized with the goat anti-rabbit Alexa-488 conjugated secondary antibody (GIBCO Life Technologies), which was applied for 75 min at room temperature in dilution 1:500 for GFAP and Iba-1 and 1:750 for Tuj-1. Staining specificity was confirmed by omission of the primary antibody. Sections were mounted with VECTASHIELD HardSet mounting medium with DAPI (4′,6-diamindino-2-phenylindole dihydrochloride; Vector Laboratories, Burlingame, CA, USA), and visualized with a Nikon E600 epifluorescent microscope equipped with Coolsnap ES2 monochrome camera (Nikon Instrument Inc.) or with Leica TCS SP8 confocal laser scanning microscope (Leica Microsystems, Buffalo Grove, IL, USA).

### Activated astrocytes quantification

Astrocyte activation was assessed using GFAP immunostained OHCs. Four to seven OHCs obtained from at least four different animals were analyzed per group. Images of immunostained OHCs were acquired using a Nikon E600 epifluorescent microscope equipped with the Coolsnap ES2 camera (Nikon Instrument Inc.) and 10× objective. For each OHC, three non-overlapping images (area 670 μm × 898 μm) were taken within the CA1 region (74) and only a small portion of the CA1 region was omitted between the images to avoid bias in which visual fields were chosen. All images were captured at identical settings. Using NIH ImageJ software (NIH, Bethesda, MD, USA), images were digitalized. In the CA1 region, GFAP mean pixel intensity (MPI) was recorded and averaged for each section (89). Data are expressed as GFAP fluorescent intensity normalized to the corresponding sham values.

### Live microglia imaging

Live microglia imaging at 4 and 24 h post-injury was performed using the microglia specific marker isolectin B-4 (IB4) isolated from *Griffonia simplicifolia* conjugated to FITC (Sigma Aldrich). According to the manufacturer’s instructions, a stock solution of FITC-IB4 was prepared in 0.9% sodium chloride solution at 1 mg/ml. Four hours prior to imaging, media containing 2 μM PI and 5 μg/ml of IB4 was applied to the OHCs (73, 90). Images were acquired under identical conditions using a Nikon Eclipse TE2000-U upright fluorescent microscope (Nikon Instrument Inc.) at 20× objective.

### Quantification of activated and total number of microglial cells

Iba1 immunostained OHCs were used to quantify activated microglial cells. Five to nine OHCs obtained from at least three different animals were analyzed per group. Images of immunostained OHCs were acquired using a Nikon E600 epifluorescent microscope equipped with the Coolsnap ES2 camera (Nikon Instrument Inc.) and 20× objective. Similar to GFAP quantification, three non-overlapping images (area 450 μm × 335 μm) were taken within the CA1 region (74), and only a small area of CA1 region was not analyzed. Using acquired images and NIH ImageJ software (NIH), an observer blinded to the exposure groups quantified the total number of activated microglia, determined by their rounded appearance with few to no cytoplasmic processes, as well as the total number of resting, ramified microglia (74). The counts obtained from three different images per section were averaged. Results are expressed as a percentage of activated microglia or total number of microglia in the analyzed ROI.

### Statistical analysis

The statistical analyses of percent area of PI staining above the threshold accounted for the effects of section, well, and plate. In addition, analyses accounted for potential correlations between percent of area assessments. The outcome of interest in our data analysis was the percent area >2%. Since the distribution of the outcome was highly skewed we used natural log transformation leading to an approximately symmetric distribution. This variance stabilizing transformation justifies the use of linear model theory with the log-transformed outcome. We performed three regression analyses, one for each hippocampal region (CA1, CA3, and DG). Each linear mixed effect regression used the same set of predictors: the random effect of plate, the random effect of well, the random effect of section, the fixed effect of experimental group (high-blast, low-blast, low-vibration control, high-vibration control, sham, and incubator control), the fixed effect of time point, and the interaction between experimental condition and time. Our model also accounted for repeated measures within each section with unstructured correlation, which accounted for the over time correlation structure and different variances at each time point. To lower the number of false discovery findings, we decreased the cutoff for statistical significance from the traditional 5 to 1%. Data are presented as model predicted values with the confidence interval.

For the LDH assay, astrocyte and microglia quantification, statistical significance between high-blast, low-blast, and sham-injured OHCs was evaluated by one-way analysis of variance (ANOVA) at a confidence level of α = 0.05 with a Tukey’s *post hoc* test. Data are presented as mean ± SEM.

The reported *n* values for PI and LDH uptake analyses refer to the number of wells that were analyzed. For PI measurements, each well was equivalent to 4–6 slices, while for LDH measurements only wells with 5 slices in each were used. For activated astrocytes and microglia analyses, *n* values refer to the number of slices that were quantified per experimental group.

## Results

### Maintenance of OHCs

**Figure 3:**
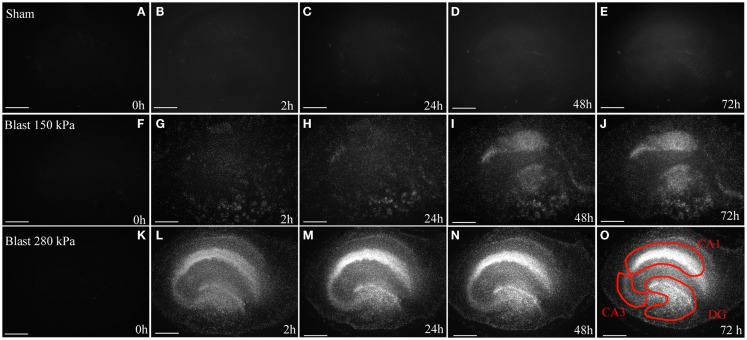
**Cell death in OHCs after blast exposure**. Following the 8 DIV recovery period from dissection, OHCs were exposed to a 150 kPa (low) or 280 kPa (high) blast overpressure or were sham-injured. **(A–E)** Representative micrographs of sham OHCs over a time course of 72 h, demonstrating low levels of dead PI-stained cells (white) throughout the experiment. In OHCs exposed to a low **(F–J)** or high blast **(K–O)** overpressure, dead cells (white) were observed as early as 2 h following injury and the damage intensified at later time points. The CA1, CA3, and the DG hippocampal regions [outlined in red in **(O)**] appear particularly vulnerable to the blast in both high and low groups. Scale bars 500 μm.

**Figure 4:**
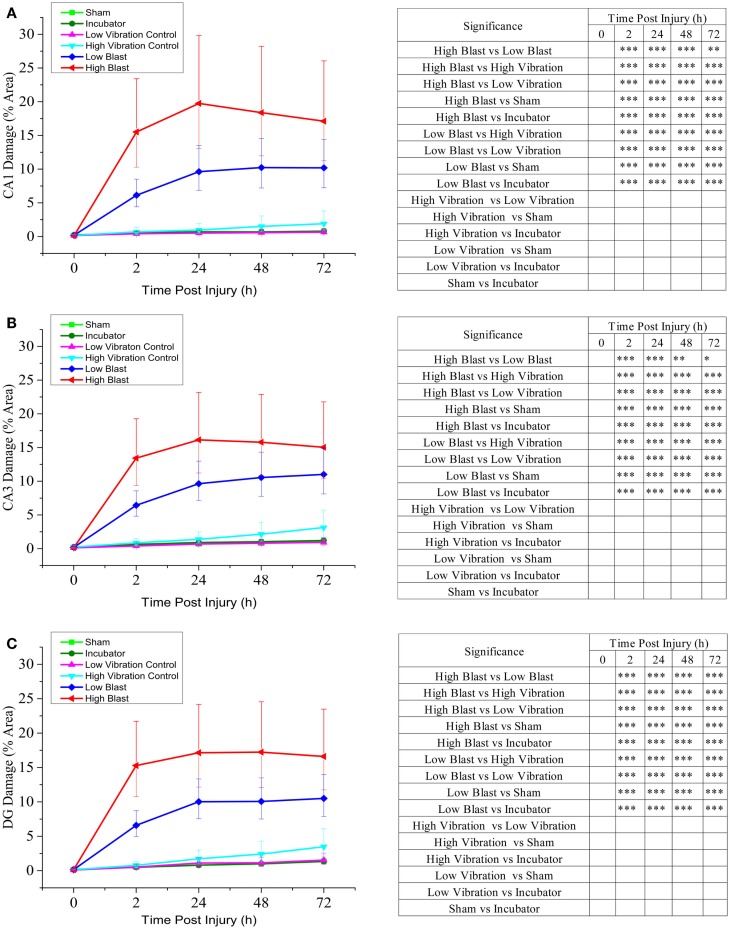
**Cell death quantification in CA1, CA3, and DG hippocampal regions following blast injury**. OHCs were exposed to overpressures of 150 kPa (low; *n* = 29) and 280 kPa (high; *n* = 13–17) and cell death was assessed at multiple time points following blast exposure using PI uptake assay. Data from the blasted groups were compared with the following control groups: sham-injured OHCs (*n* = 35–38), incubator controls (*n* = 38–39), low-vibration controls (*n* = 6–7), and high-vibration controls (*n* = 5). Quantitative analysis of blast-evoked cell death was performed by measuring the percent area of CA1 **(A)**, CA3 **(B)**, and DG **(C)** regions with PI staining above the threshold. Results are expressed as values and confidence interval predicted by the linear statistical model. *P < 0.05; **P < 0.01, ***P < 0.001.

**Figure 5:**
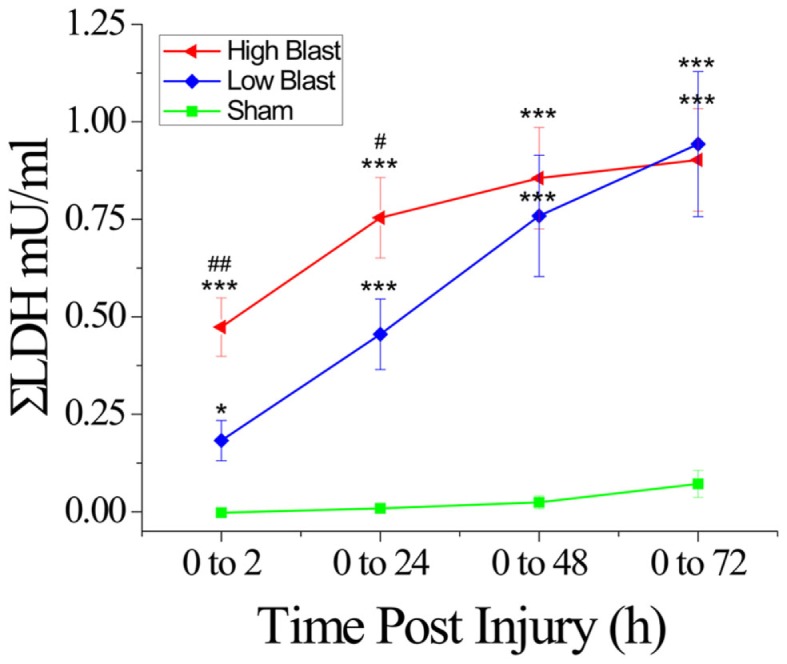
**Lactate dehydrogenase release in response to blast damage**. Measurements of LDH released into the culture medium up to 72 h following exposure to high or low blast overpressures indicated a significant difference in the amount of LDH released into the medium between high and low blast at 2 and 24 h, and a significant difference between blasted sections and shams at all time points post-injury. *P < 0.05, ***P < 0.001, blast compared to sham-injured OHCs, ^#^*P* < 0.05, ^##^*P* < 0.01, high- compared to low-blast group. *n* = 5–8 wells per each experimental group (each well equivalent to 5 slices).

Organotypic hippocampal slice cultures’ ultrastructural organization was well preserved throughout the culture period as evaluated by phase-contrast microscopy and cresyl violet staining (Figure 2). In accordance with previously described characteristics of interface slice cultures (91), after 8 DIV OHCs thinned due to the spread of tissue, but the major hippocampal regions (CA1, CA3, and DG) were well conserved with clearly visible boundaries (Figures 2A,B). Maintenance of typical hippocampal cytoarchitecture in OHCs was also demonstrated with cresyl violet staining at 8 DIV (Figures 2C,D). In addition, using a PI uptake assay and serial imaging at 1, 5, and 8 DIV, we have confirmed results from previous studies (72, 73) that 7 days is a sufficient period to allow OHCs to recover from procedure-related cellular degeneration (Figures 2E–G). Moreover, the low level of PI staining (Figures 3 and 4) and LDH release (Figure 5) observed in incubator and sham controls throughout the experiment confirmed good vitality of OHCs in our experiments.

### Assessment of blast-induced cell death in OHCs by PI uptake

Low levels of PI fluorescence at 8 DIV indicated very small levels of cell death in OHCs prior to blast exposure (Figure 3). Both incubator and sham controls maintained low levels of PI fluorescence throughout the experiment (Figures 3 and 4), although a small increase in PI staining was observed over time as the result of slight deterioration in OHCs during prolonged culturing (Figures 3 and 4). In addition, similar low levels of PI staining were observed in low- and high-vibration controls (Figure 4), demonstrating that shock tube vibration did not induce significant mechanical damage and cell death. Increased PI staining was observed as early as 2 h post-injury for both low- and high-blast groups (Figures 3 and 4), and cell death was most prominent in the CA1, CA3, and DG hippocampal regions (Figure 3). Both the low- and high-blast groups had a significantly higher percent area of PI staining within CA1, CA3, and DG regions compared to control groups at all analyzed time points following injury (Figure 4). Moreover, blast-evoked cell death was dose-dependent and the percent area of CA1, CA3, and DG regions occupied with the PI staining was significantly greater in the high-blast group than in the low-blast group at all analyzed time points (Figure 4). In addition, the low- and high-blast groups demonstrated different kinetics of the blast-evoked cell death. OHCs exposed to the low blast exhibited a slower increase in cell death compared to OHCs exposed to the high blast, which demonstrated dramatic increase in cell death already at 2 h post-injury. In addition, percent area of PI staining above the threshold within ROI reached the plateau between 24 and 48 h post-injury in the low-blast group, while in the high-blast group plateau was reached at 24 h post-injury. A slight decrease in cell death at 24 h following high blast is attributed to the detachment of dead cells.

### LDH efflux as a measure of blast-evoked cell death in OHCs

Measurement of LDH release into the culture medium at 2, 24, 48, and 72 h following blast exposure demonstrated changes in OHCs viability (Figure 5), similar to those assessed by the PI uptake assay (Figure 4). In the high-blast group, the highest rate of cell death occurred within 2 h following injury, while the low-blast group cell death rate gradually increased up to 48 h following injury (data not shown). Additionally, cumulative LDH release in the low- and high-blast groups was significantly greater than in sham controls at all analyzed time points (Figure 5). Furthermore, there was a significant difference in the cumulative LDH release between the high- and low-blast groups at 2 and 24 h post-injury (Figure 5).

### Vulnerability of neurons in OHCs to blast exposure

**Figure 6:**
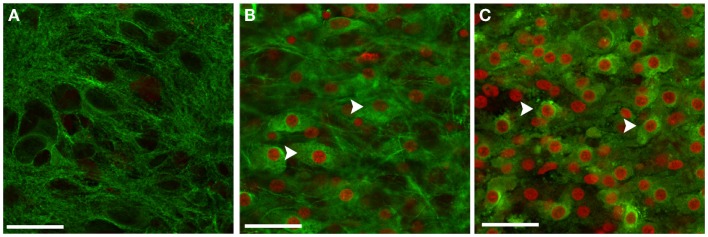
**Neurons in OHCs were particularly vulnerable to blast injury**. Representative confocal images of CA1 region of sham-injured **(A)**, low **(B)**, and high blast-exposed OHCs **(C)** at 72 h following injury. Sections were co-stained against neuronal marker Tuj1 (green) and PI (red). Confocal images with the overlay of PI and Tuj1 staining demonstrated good viability of neurons in the sham OHCs **(A)**, and significant number of killed neurons in blast-exposed OHCs **(B,C)** that co-expressed Tuj-1 and PI (arrows). Scale bars 25 μm.

Following blast exposure, cell death visualized by PI staining was mainly present in the hippocampal neuronal layers including pyramidal cells of the CA1 and CA3 regions, and granule cells of the DG (Figure 3). Co-staining with PI and neuronal marker Tuj1 at 72 h post-injury further demonstrated that the majority of blast-killed cells were neurons that co-stained for PI and Tuj1 (Figure 6).

### Astrocyte activation following OHCs’ blast exposure

**Figure 7:**
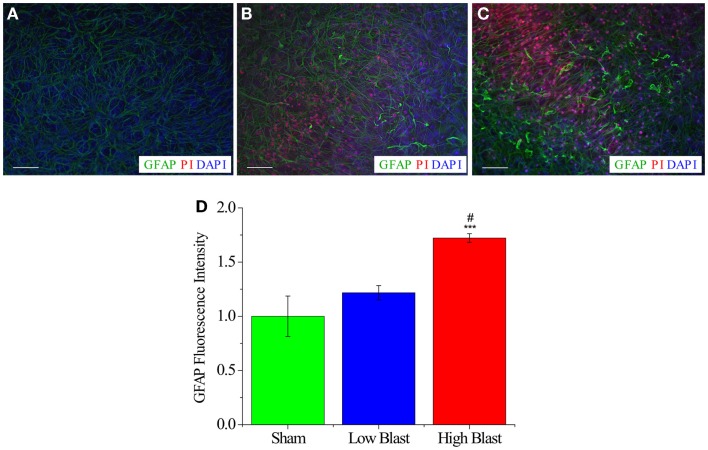
**Astrocyte activation in blast-exposed OHCs**. Representative images of CA1 region from sham-injured **(A)**, low-blast **(B)**, and high-blast OHCs **(C)** that were fixed at 72 h following blast exposure and stained with anti-GFAP (green), PI (red), and the nuclear counter stain (DAPI). **(A)** OHCs maintained low level of astrocyte activation and PI staining at 72 h following sham injury. Activated astrocytes, as visualized by increased GFAP expression, hypertrophy, and thicker processes, were observed both in low- **(B)** and high-blast **(C)** groups at 72 h following blast exposure. **(D)** Quantification of GFAP staining demonstrated significant increase in GFAP MPI in OHCs exposed to high-blast compared to sham-injured OHCs (***P < 0.001) and OHCs exposed to the low blast (^#^*P* < 0.05). Scale bars 50 μm.

Activated astrocytes, indicated by increased GFAP expression and cellular hypertrophy, were observed in high- and low-blast groups at 72 h post-injury (Figure 7). Quantification of GFAP staining demonstrated significant increase of GFAP MPI in the high-blasted group compared to the sham-injured OHCs (*P* < 0.001). Moreover, high-blasted OHCs demonstrated more vigorous astrocyte activation compared to the low-blasted OHCs (*P* < 0.05), implying blast-evoked dose-dependent astrocyte activation (Figure 7). Low-blasted OHCs demonstrated an increase in GFAP MPI compared to sham controls; however, this effect did not reach statistical significance (Figure 7). A small number of GFAP-labeled astrocytes co-localized to PI staining at this time point (Figure 7).

### Blast-evoked microglial death and activation in OHCs

**Figure 8:**
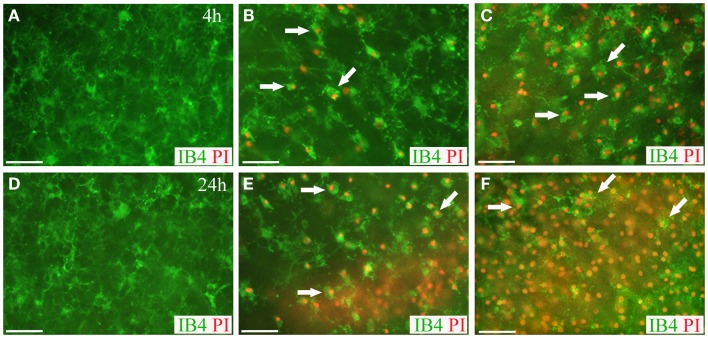
**Live microglia imaging in OHCs following blast exposure**. Microglial cells in OHCs were labeled with IB4 (green) and images of CA1 region were captured at 4 h **(A–C)** and 24 h **(D–F)** following sham **(A,D)** or blast injury **(B,C,E,F)**. OHCs maintained low level of microglial activation and PI staining (red) at 4 h **(A)** and 24 h **(D)** following sham injury. Dead microglial cells that were co-labeled with IB4 and PI (arrows) were observed in low- **(B)** and high- **(C)** blast group at 4 h post-injury. Even more prominent microglial death was detected in low- **(E)** and high- **(F)** blasted OHCs at 24 h post-injury. Activation-induced change in microglia morphology from ramified to rounded was also observed in blasted OHCs **(B,C,E,F)**. Scale bars 50 μm.

**Figure 9:**
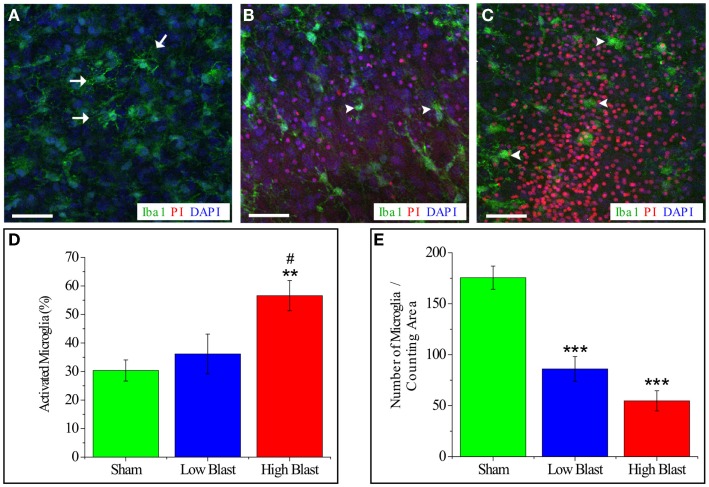
**Quantification of activated microglia and total microglia number per counting area in blast-exposed OHCs**. OHCs were fixed at 72 h post-injury and stained with Iba1 (green), PI (red), and DAPI counter stain (blue). Representative confocal images of CA1 region of sham-injured **(A)** and blasted OHCs **(B,C)**. Sham-injured sections **(A)** showed ramified, resting microglia (arrows). Low-blast **(B)** and high-blast **(C)** OHCs demonstrate increased number of activated, rounded, amoeboid microglia (arrowheads). Scale bars **(A–C)** 50 μm. **(D)** Quantification of activated microglia within ROI in CA1 area revealed significantly higher percentage of activated microglia in high-blast OHCs compared to sham controls (**P < 0.01) and compared to low-blast sections (^#^*P* < 0.05). **(E)** Quantification of total number of microglial cells per counting area in CA1 region demonstrated significant decrease in OHCs exposed to low (***P < 0.001) and high blast (***P < 0.001) compared to the sham-injured OHCs. *n* = 5–9 sections per each experimental group.

Live-cell imaging of IB4-labeled microglial cells at 4 and 24 h post-injury reveled that some of the microglial cells were co-labeled with PI implying blast-evoked microglial death (Figure 8). In addition, while the majority of the IB4-labeled microglial cells in the sham OHCs appeared as ramified – resting microglia, microglial cells in low- and high-blasted OHCs possessed mainly rounded morphology pertinent to their activation (Figure 8). Activation of microglial cells following blast exposure was also confirmed in Iba1 immunostained OHCs at 72 h post-injury (Figure 9). Quantification of Iba1 immunostained, activated microglial cells at 72 h post-injury in the CA1 ROI indicated that 57 ± 5% of microglial cells in the high-blast group were activated, which was significantly higher than 30 ± 4% of activated microglial cells in sham controls (*P* < 0.01; Figure 9). At the same time point, 36 ± 7% of microglial cells were activated in the low-blast group, which was also significantly different compared to the high-blast group (*P* < 0.05; Figure 9). The low-blast group demonstrated a trend toward increased percentage of activated microglial cells compared to the sham-injured OHCs (36 ± 7 vs. 30 ± 4%), although this difference did not reach statistical significance (Figure 9). Compared to the earlier time points following blast exposure, at 72 h post-injury a smaller number of microglial cells co-localized with the PI staining (Figures 8 and 9). However, at 72 h post-injury the total number of microglial cells per counting area in low- (*P* < 0.001) and high-blasted (*P* < 0.001) OHCs was significantly smaller compared to the sham-injured OHCs (Figure 9).

## Discussion

With the increasing incidence of bTBI among military personnel and civilians (1, 2, 92–94), it is necessary to better understand and depict the mechanisms of blast-evoked neurodegeneration in order to produce effective treatment strategies. The intent of this experimental study was to evaluate brain tissue cell death following exposure to blast shockwaves. To that end, a number of experimental considerations were devoted to limiting exposures to pure shockwave overpressure including placing specimens off axis from the shock tube, quantifying damage attributable to system vibration, and obtaining high-speed videos of samples (cell culture dishes with the inserts) to ensure a lack of displacement. Accordingly, samples were not exposed to exhaust gases from the shock tube, vibration shams did not demonstrate additional damage beyond the normal shams or incubator controls, and high-speed video did not reveal cell culture dish displacements during shockwave exposures. However, the authors acknowledge that some component of tertiary damage may have contributed to the tissue injury reported in this study. As such, it remains possible that acoustic impedance mismatch between the different materials (e.g., air, well, medium) may have resulted in inertial loading that may have led to mechanical deformation (i.e., strain) of the tissue samples. Unfortunately, it was not possible to quantify this aspect, as inclusion of pressure transducers inside the well or changing the setup to enable high-speed video of the uncovered dish with the insert would have changed the end conditions and resulted in data not applicable to the current setup. However, acoustic impedance differences also exist in the human condition (e.g., air, skin, cranium, dura mater, etc.) and the exact mechanism of brain tissue damage during blast has not been conclusively determined. For example, studies have hypothesized that injury occurs due to high rate/small magnitude tissue strains (95), compression followed by rapid expansion of brain tissues due to the shockwave pressure spike (96), or overpressure-induced axonal injury in the first few milliseconds of exposure prior to significant head motions (97). All of these mechanisms include mechanical deformation of brain tissues, although strain magnitudes, rates, and types have not been conclusively outlined.

So far, several different mechanisms have been implicated in primary blast-induced bTBI that are not necessarily mutually exclusive (19). Previous work has highlighted the role of head rotational acceleration in producing brain injury during blast (20). Moreover, using numerical hydrodynamic simulations, Moss and colleagues (17) have discovered that skull flexure might be implicated in bTBI. In addition, several studies that implemented rat models with whole-body or local (chest) exposure to the blast overpressure, suggested that activation of the autonomous nervous system, sudden pressure-increase in vital organs such as lungs and liver, pulmonary injury, and activation of neuroendocrine-immune system are among mechanisms that significantly contribute to the brain injury following blast exposure (15, 18, 98). Although it is hard to compare results obtained from different bTBI models, prior *in vivo* studies by our group combined with the present outcomes suggest a direct role of overpressure exposure in bTBI. For example, statistically significant cognitive deficits, emotional changes, and structural evidence of damage using diffusion tensor imaging and histological examination were evident following exposure of SD rats to shockwave overpressures of 100 and 450 kPa (25). That study incorporated off axis exposures to mitigate effects of shock tube exhaust gas, body protection to prevent lung and heart injury, and constrained the rodent head against significant head rotational accelerations. Therefore, injuries and associated behavioral deficits were directly attributable to shockwave overpressure and not head rotational acceleration, skull flexure, thoracic mechanisms, or pulmonary ischemia. The concept of direct propagation of the shockwave through the cranium was proven in theory by our group using a post-mortem human subject model (24). That study determined that shockwave overpressures propagate intact through the skull and maintain the characteristic Friedlander waveform inside the cranium without significant loss of peak overpressure magnitude for blast exposures from the frontal and lateral directions. Similarly, it has been demonstrated that the shockwave passes almost unchanged through the rat (99) and pig skull (21, 23). Moreover, studies that utilized finite element modeling (FEM) of blast waves applied to human head models demonstrated the potential for blast waves to enter the cranium directly (97).

Present studies were conducted in an OHC-based *in vitro* bTBI model that enabled us to further evaluate isolated effects of a blast exposure on neurons and glial cells with native brain tissue organization preserved. Our data demonstrated that blast exposure can directly cause neuronal damage and death, and to a lesser extent glial loss. In addition, blast exposure resulted with the glial activation in OHCs. As discussed above, several studies demonstrated the ability of the blast wave to pass through the skull and cause direct effects on the brain tissue (21, 23–25, 97, 99), suggesting that propagation of the blast wave through the blood vessels or CSF are not the main mechanisms of bTBI. Thus, OHC-based models are suitable to study bTBI, even without the presence of blood and CSF circulation. Previously, validity of OHCs for *in vitro* modeling of different neurodegenerative diseases was confirmed in numerous studies [reviewed in Ref. (47, 49)]. Additionally, a similar OHC-based *in vitro* bTBI model recently reported blast-evoked cell death in hippocampal tissue (28, 29), which is in agreement with our data. However, blast-evoked cell death was less prominent in those studies and did not attain statistically significant differences from shams until 4 days post-injury. In contrast, cell death was observed in the present study as early as 2 h post-shockwave exposure and progressed over the course of several days. Differences in outcomes between present results and prior work is likely attributable to differences in experimental model design. In prior work, culture inserts with OHCs were sealed inside a medium-filled bag that was submersed in a water-filled sample receiver. In the present study, dishes containing serum-free medium and Millicell inserts with OHCs were sealed inside sterile plastic pouches and placed in air below the shockwave tube to ensure perpendicular exposure. These differences may partially explain lower injury severities from more severe exposures (424 kPa, 2.3 ms) in studies by Effgen and colleagues (28, 29).

Other studies have demonstrated a similar time course of cell death and damage to the present results. Damage and death of hippocampal neurons were detected at 2 h after exposure to a short-lasting impulse noise (100, 101). In addition, neuronal degeneration and loss were observed in the hippocampus within a few hours following shockwave exposure in several *in vivo* studies (15, 34, 40, 102). It has been suggested that these ultrastructural and biochemical impairments in the hippocampus correlate with the cognitive deficits observed in rats shortly following blast exposure (15, 34, 102). In clinical studies, memory impairment in blast victims was also recorded already at 72 h following exposure (103), which was likely result of the hippocampal damage.

In our *in vitro* bTBI studies, rapid onset of cell death was visualized via PI staining and increased LDH release. Damage to the integrity of the plasma membrane must be sustained for PI to enter the cell or LDH to be released into the culture medium (44, 77, 78). Although exact mechanisms of blast-evoked cell death are currently unknown, our data suggest that mechanical damage caused by shockwave exposure results in cell membrane damage that ultimately leads to the increased bidirectional transport of molecules and cell death. Accordingly, increased cell permeability was observed following shockwave exposure in dorsal root ganglion (DRG) (104) and human neuroblastoma cells (27). This is the first study to report similar effects on brain tissue.

The present study incorporated two overpressure magnitudes (147 ± 18 and 278 ± 22 kPa) to quantify the dose effect of increasing shockwave overpressure. These applied peak overpressure magnitudes are within the range of overpressures that were previously tested in rat bTBI models by our (25) and other groups (16, 33, 105), and have resulted in neurodegenerative changes and behavioral impairments. Moreover, these overpressure magnitudes are comparable to the survivable blast overpressures experienced by soldiers in the field (106, 107). Quantification of PI staining demonstrated a significant increase in cell death following exposure to 280 kPa compared to 150 kPa at all analyzed time points following injury. Dose-dependent relationships between the blast overpressure magnitude and neuropathological changes, behavioral deficits (16, 25, 108, 109), and mortality rate (109, 110) were previously reported in several *in vivo* bTBI studies, although other *in vivo* studies did not demonstrate a similar dose-dependent relationship (102, 111, 112). A possible explanation for these conflicting *in vivo* data is that blast-induced brain injury severity does not correlate with peak pressure alone and it likely depends on other factors including positive phase duration and impulse (25, 29, 36, 95, 106, 107).

Following blast exposure, we observed PI-stained dead cells mainly in neuronal CA1, CA3, and DG hippocampal regions. This pattern of cell death suggested that neurons are predominantly affected by blast exposure. Further, at 72 h post-injury, the majority of blast-killed cells were neurons as demonstrated with the Tuj1 and PI co-staining. To a lesser extent, PI-stained cells were co-labeled with markers for microglial cells (Ib4 and Iba1) and astrocytes (GFAP), implying that glial cells are more resistant to blast exposure. Co-labeling of PI with microglia markers IB4 or Iba1 demonstrated a higher number of dead microglial cells at earlier time points following blast exposure. We speculate that these dead microglial cells detach from OHCs since at 72 h post-injury a smaller number of microglial cells were co-labeled with PI. Similarly, at 72 h post-injury only a small number of GFAP immunostained astrocytes were co-labeled with PI. However, we cannot exclude the possibility that astrocytes die in a higher number at the earlier time points following blast exposure. In correlation with our data, *in vivo* studies (15, 20, 34, 39, 40, 51, 102) reported significant hippocampal neuronal degeneration and loss following blast overpressure exposure. Moreover, our data suggest that neurons within CA1 hippocampal region are somewhat more vulnerable to the blast exposure than neurons in DG and CA3 regions. In accordance with our results, Effgen at al. reported the highest percent of cell death in CA1 region in OHCs exposed to the pressure level 9 (29). Similarly, significant loss of CA1 pyramidal neurons followed by delayed cell loss in the DG was observed in OHCs exposed to oxygen–glucose deprivation (113).

Our findings demonstrated that glial cells were activated in OHCs following blast exposure. Astrogliosis, the activation and increase in astrocytes in the central nervous system, has been reported in response to increased neuronal damage in different neurodegenerative diseases [reviewed in Ref. (114–117)]. In addition, astrocytes hypertrophy and increased expression of GFAP were observed in response to blast exposure in numerous animal models (36, 39, 102, 118–121). Several studies suggested the importance of astrogliosis in regulation of inflammation (122–124) and neuroprotection/recovery of damaged tissue (125–127). However, other studies have linked astrogliosis to an increased incidence of apoptosis and inhibition of neural growth following injury due to the generation of a glial scar (128–130). At 72 h post-injury activated, hypertrophic astrocytes were observed both in low- and high-blast groups. However, astrocyte activation was more prominent in the high-blast group and the average GFAP MPI in this group was significantly higher than in sham and low-blast groups. The dose-dependent activation of astrocytes that is implied by our results has been previously suggested in animal bTBI models (109, 121), demonstrating similarity to our *in vitro* model.

Alongside astrogliosis, microglial activation has been strongly associated with various neurodegenerative disorders, including blast (32, 38, 120) and non-blast TBI (131), Parkinson’s disease (132, 133), and Alzheimer’s disease (134, 135), among many others. Microglia are the primary immune cells of the central nervous system and their activation results in the release of various proinflammatory factors, including free radicals, cytokines, and proteinases (134, 136–138). The majority of evidence suggests microglial activation following a nervous system insult ultimately results in further neurodegeneration (38, 135, 139, 140); however, it remains controversial whether microglial activation can potentially be beneficial and protective under certain circumstances (132, 138, 141–143). Previous studies of microglial cells in OHCs have demonstrated that these cells are highly activated at the beginning of the culturing period and that the number of activated microglial cells gradually decreases from 3 DIV (75). At 7 DIV, the majority of microglial cells return to the resting phenotype, but a certain percentage of activated microglia is still present (74–76). In our studies, we have observed about 30% of activated microglial cells in sham OHCs at 72 h post-injury. In agreement with our results, Billingham and colleagues (76) have observed about 25% of activated microglial cells in control OHCs at 7 DIV, while Czapiga and Colton (68) detected in OHCs about 20% of activated microglia with phagocytic activity at 10 DIV. Despite the existence of a heterogeneous microglia population throughout the culturing period, it has been reported that OHCs are a suitable model to study the microglial inflammatory response (84). Accordingly, dynamic transformation of microglial morphology upon activation from ramified and highly branched to amoeboid was reported in several studies conducted in OHCs (68, 74, 75, 144–146). Following OHC exposure to the low and high overpressures, we observed an increased number of rounded microglial cells, consistent with their activation. This effect was more pronounced in the high-blast group in which the percentage of activated microglial cells was significantly higher compared to the sham and low-blast group at 72 h post-injury. In our studies, we have also observed microglial cells that were co-labeled with PI and IB4. Some of these co-labeled cells may illustrate phagocytic clearance of dead cell nuclei by microglial cells (73). However, our data suggest that the majority of these PI and IB4 co-labeled microglial cells represented dying cells since the total number of microglial cells per counting areas within the CA1 region was significantly decreased in the blasted groups at 72 h post-injury. We speculate that microglial loss observed in our studies was caused not only directly by the blast exposure but also indirectly by the blast-induced microglia over-activation. Previously, it has been shown that over-activation of microglial cells can lead to their death (147). Similar to data from our *in vitro* model, microglial activation was observed following animal shockwave exposure (32, 40, 120). Furthermore, post-mortem analysis of military veterans suffering from a bTBI further confirms activation of microglia (20), validating our *in vitro* model as representative of *in vivo* scenarios. It is controversial whether this microglial activation observed *in vivo* following blast exposure is the direct effect of a shockwave, or if it is caused by an increase in the blood–brain barrier permeability and allocation of inflammatory mediators from circulation (32). However, our studies demonstrated that microglial activation in the brain tissue is the direct effect of the blast exposure. Moreover, there have been conflicting reports in bTBI models regarding microglial activation response time. In accordance with our data, Turner and colleagues (148) reported microglial activation at 72 h post-blast injury. However, several groups reported a delayed microglial response at 7 days post-blast (121, 149) or 2-weeks post-blast (40). The 72 h activation of microglia reported in this study is likely correlative with the early onset of cell death following blast injury assessed by PI and LDH assays. We observed the majority of activated microglial cells around the dead neurons in the CA1 region. This corroborates with previous studies that have reported microglial migration and accumulation around damaged neurons in OHCs and suggested their role in clearing away dead cells and debris (73, 146).

Overall, our data provide insight into the cellular mechanisms of neurodegeneration in response to blast exposure and have confirmed direct effects of a blast on neuronal and glial cells. Together, these findings prove validity of our *in vitro* bTBI model for studying mechanisms underlying neurodegenerative changes following blast exposure, as well as for screening novel therapeutic modalities.

## Conflict of Interest Statement

The authors declare that the research was conducted in the absence of any commercial or financial relationships that could be construed as a potential conflict of interest. The Review Editor Joseph Long declares that, despite having collaborated with author Matthew D. Budde, the review process was handled objectively and no conflict of interest exists.
